# A Theoretical Analysis of the Effects That the Glycocalyx and the Internal Elastic Lamina Have on Nitric Oxide Concentration Gradients in the Arterial Wall

**DOI:** 10.3390/antiox14060747

**Published:** 2025-06-17

**Authors:** Yaroslav R. Nartsissov, Irena P. Seraya

**Affiliations:** 1 Department of Mathematical Modelling and Statistical Analysis, Institute of Cytochemistry and Molecular Pharmacology, Moscow 115404, Russia; irena_seraya@mail.ru; 2 Biomedical Research Group, BiDiPharma GmbH, Bültbek 5, 22962 Siek, Germany

**Keywords:** nitric oxide, convectional reaction–diffusion, blood flow, internal elastic lamina, glycocalyx

## Abstract

Nitric oxide (NO) is a well-known member of the reactive oxygen species (ROS) family. The extent of its concentration influences whether it produces beneficial physiological effects or harmful toxic reactions. In a blood system, NO is generally produced by nitric oxide synthase (NOS) in the endothelium. Then, it diffuses into the smooth muscle wall causing a vasodilatation, and it can also be diluted in a lumen blood stream. In the present study, we analyzed a convectional reaction–diffusion of NO in a 3D digital phantom of a short segment of small arteries. NO concentrations were analyzed by applying numerical solutions to the boundary problems, which included the Navier–Stokes equation, Darcy’s law, varying consumption of NO, and the dependence of NOS activity on shear stress. All the boundary problems were evaluated using COMSOL Multiphysics software ver. 5.5. The role of two diffusive barriers surrounding the endothelium producing NO was theoretically proven. When the eNOS rate remains unchanged, an increase in the fenestration of the internal elastic lamina (IEL) and a decrease in the diffusive permeability of a thin layer of endothelial surface glycocalyx (ESG) lead to a notable rise in the NO concentration in the vascular wall. The alterations in pore count in IEL and the viscosity of ESG are considered to be involved in the physiological and pathological regulation of NO concentrations.

## 1. Introduction

Nitric oxide (NO) is a well-known signal transduction radical mediating a variety of physiological processes in arteriolar tone [1]. In the physiological regulation of the cardiovascular system, NO is vital, as any abnormalities in its production or availability can accompany or even precede conditions such as hypertension, atherosclerosis, and disorders linked to angiogenesis [2]. Moreover, NO is involved in various physiological processes within the nervous and immune systems, playing a key role in behaviour regulation, gastrointestinal activity, and the defence against infections and cancerous growths [3]. This radical is produced cellularly by NO synthase (NOS) through a reaction that converts L-arginine and oxygen into citrulline and NO [4]. NOS is a complex protein with three isoforms that are homologous, although they differ in their structure, regulation and distribution: neuronal NOS (nNOS), endothelial NOS (eNOS) and inducible NOS (iNOS) [5,6]. eNOS is mostly expressed in endothelial cells, and it synthesizes NO in a pulsatile manner with synthetase activity markedly increasing when intracellular Ca^2+^ rises [5]. Then, NO diffuses into the external vessel wall and into the blood stream. Low levels of NO produced by endothelial cells causes the relaxation of vascular smooth muscle cells and consequent vasodilation [7]. These NO functions are mediated by cyclic GMP synthesized through soluble guanylyl cyclase (sGC), a heme-containing enzyme, which is directly activated by NO [8]. At the same time, NO is considered a toxic agent. In particular, it was demonstrated that NO (from macrophages or exogenously administered) primarily inhibits oxidative phosphorylation in the mitochondria of target cells [9]. This inhibition occurs because NO reversibly binds to cytochrome-C-oxidase of the mitochondrial electron transport chain [10]. In terms of a molecular mechanism, the reduction in eNOS functionality caused by substantial uncoupling [5] plays an essential role in the development of cardiovascular diseases, as it increases the synthesis of peroxynitrite (ONOO–) during episodes of ischaemia–reperfusion or infarction [11]. Therefore, when a gradient of NO concentration is established, it is crucial to maintain a balance between physiological effects and potential toxicity, and multiple regulatory mechanisms contribute to achieving a stable level of its production and distribution. Both experimental and theoretical investigations of these processes are necessary to create new strategies for therapeutic applications in clinical setting.

Various types of known mechanisms that regulate the gradients of NO concentration exist. Indeed, the simplest option is an up/down regulation of the NOS activity. It provides a direct shift in NO concentration, both in vessel walls and in surrounding tissue. Nevertheless, some additional biological aspects are also important in this case. First, a blood flow essentially contributes as a convectional component during NO concentration gradient appearance. It means that one may expect differences in the spatial–temporal distribution of NO under various flow rates in the blood vessels. A blood stream can quickly remove the synthesized NO away from the endothelium. This process essentially shifts the balance of the NO concentration, especially in a large vessel. Second, the anatomic structure of arteria is very heterogenous. It is essential to recognize that the various structural components possess distinct physical and chemical characteristics that can influence the gradient of NO concentration. In particular, the endothelium, where NO is synthesized, lies between the internal elastic lamina (IEL) [12] and the inner vessel lumen, which is lined with endothelial cells coated with a thin layer of endothelial surface glycocalyx (ESG) [13]. It is tempting to assume that NO movement is impeded by the influences of both IEL and ESG. Additionally, one should note that if the internal elastic lamina is usually considered a regular barrier of a molecule’s diffusion [14], then EGS can even directly regulate NO levels [15,16]. At the same time, ESG consists of proteoglycans, glycosaminoglycans (GAGs), and glycoproteins [15] in a ratio forming a final layer viscosity. Lowering or removing particular constituents influences the viscosity of ESG, thereby impacting its diffusive permeability. Thus, the question of the direct permeability of structures to NO is still a subject of discussion.

When examining the development of NO concentration gradients, it becomes impossible to distinguish between the impacts of variations in blood flow and changes in the characteristics of the medium because multiple simultaneous processes play a role in determining the final distribution. An elevation in blood flow velocity leads to an increase in shear stress and eNOS activity; however, this effect may be reduced if the medium’s diffusion permeability is lowered. To find a solution for this challenge, the application of numerical mathematical modelling is used to reveal the separate contributions. The analysis of the NO concentration gradients in a blood vessel wall is fulfilled using mathematical modelling of a 3D digital phantom of a short segment. Initially, the phantom is created on the base of known experimental data and common anatomic structures. Then, the boundary problem for a convectional reaction–diffusion equation is solved in all considered domains. The convection velocity field is obtained using the solution of the Navier–Stokes equation for the lumen of a blood vessel and Darcy’s law for other areas of the phantom. Through the theoretical approach provided in the present study, it is possible to directly gauge how the interplay of various physico-chemical factors affects the NO concentration gradient. The principal hypothesis under examination is whether the properties of arterial structural elements exhibit a significant effect in relation to the activity level of eNOS.

In the present study, we show that not only the activity of NOS but also the diffusive properties of IEL, ESG, and blood flow velocity have an essential influence on the NO gradients in a vessel wall. A higher permeability of IEL and a lower permeability of ESG are necessary to form a larger gradient of NO under the same eNOS activity. These theoretical conclusions provide an innovative explanation for the experimentally identified link between ESG properties and NO levels in the walls of blood vessels [15], based on direct variations in ESG viscosity. The diffusive barrier related to ESG serves as a backdrop for the NO concentration gradient within the smooth muscle arterial wall, even though shear stress still stimulates eNOS activity. The absence of a diffusive barrier results in the disappearance of the NO gradient, attributed to its intensive access to a blood flow that has a high consumption rate, and this effect is likely to be intensified by an assumed lack of eNOS, which is affected by shear stress.

## 2. Materials and Methods

**Figure 1:**
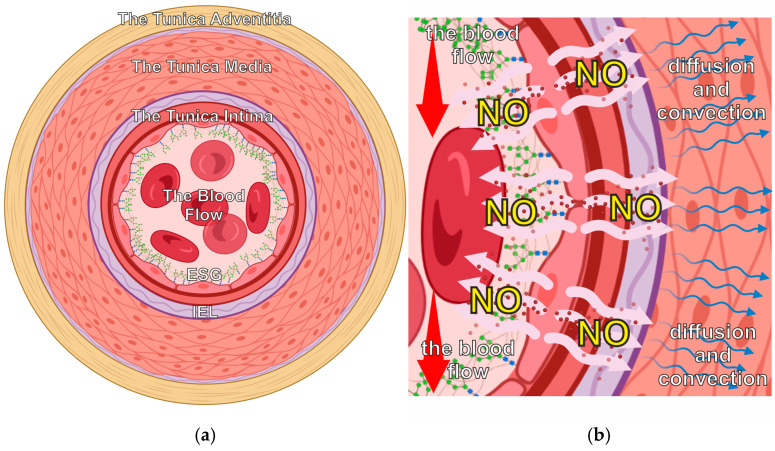
The structure of arteria. (**a**) The main areas that form a blood vessel are the lumen including the blood flow and three tunics (intima, media, adventitia). The last ones include well-described anatomical elements [17]. ESG is a part of the lumen, and it lines the internal boundary of the endothelium. IEL covers the external boundary of the tunica intima, being a part of it. (**b**) A schematic illustration of NO production in the endothelium and its farther convectional reaction–diffusion into the tunica media and the blood flow. Created in https://BioRender.com (accessed on 24 January 2025).

In order to start analyzing the concentration gradients of NO, it is crucial to first examine the layered configuration of an arterial segment. Remarkably, all types of arterial vessels are composed of well-established anatomical elements [17]. There are four structured areas that form a blood vessel (Figure 1a), and they all take part in the dynamic process of forming a gradient for NO concentration.

Various simultaneous physical processes contribute to the establishment of NO concentration distribution within the layers. They include reaction–diffusion and convection, and they are merged in various ways depending on the area (Figure 1b).

The evaluation method for the NO concentration gradient is determined by the configuration and type of processes under consideration, as detailed below. The formulation of the mathematical model comprises three interrelated steps: (i) the creation of a three-dimensional digital phantom for the area; (ii) the evaluation of the convection velocity field; and (iii) solving boundary problems linked to convection and reaction–diffusion in each area. The first stage focuses on crafting a digital design that relies on logical operations and Boolean algebra. This can be produced through a range of CAD applications (AutoCAD 24, ABViewer 14, etc.) or by utilizing geometric tools found in numerical software. The next stage pertains to the evaluation of the velocity field related to convection processes. As already mentioned above, it is obtained in a blood stream and in the surrounding layers using the Navier–Stokes equation and Darcy’s law, respectively. The convection velocity values are further included in the reaction–diffusion equations to estimate the NO concentration gradients. Moreover, addressing the Navier–Stokes equation facilitates the acquisition of wall pressure and shear stress, which are utilized in the context of Darcy’s law convection and analyzing the dependence of eNOS activity, respectively. Finally, the gradient of the NO concentration can be estimated using the boundary problem for the convectional reaction–diffusion equation. All these stages will be consequently described in the subsections below. As already stated above, in order to obtain the numerical results mentioned above, a digital phantom of the considered area should be initially designed. In the next section, this process will be described in detail.

### 2.1. The Creation of an Arteria 3D Digital Phantom

The object considered for modelling in the present study is a short length of a small arteria. The choice is based on several factors. First, the width of the artery must be greater than that of capillaries [18] but less than the dimensions of vessels supplied by vasa vasorum [19]. Second, the length of the vessel needs to be adequate to avoid any bifurcation points at that diameter [20].

**Figure 2:**
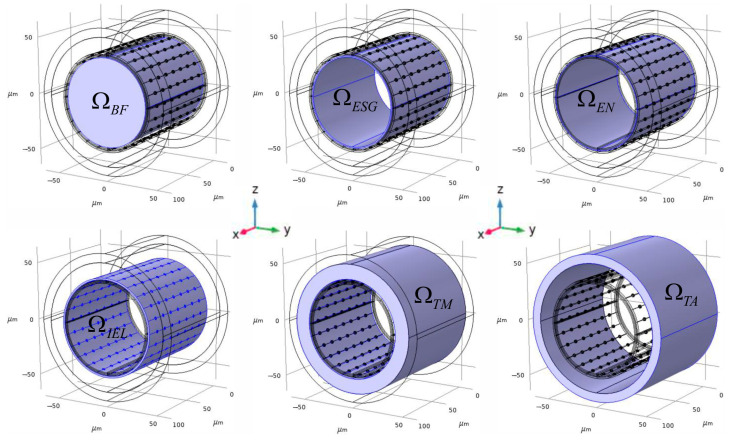
The 3D digital phantom of an arteria. The represented geometrical forms indicate the anatomic structures of the blood vessel as the set of full-body cylinders, including one after another. According to the final Form Union procedure, the cylinders will form the domains with a pipes’ body structure. The 3D digital phantom has been represented for the arteria of 80 μm diameter and for the case of a high IEL fenestration.

The phantom of the considered area is designed as a set of inserted shot pipes surrounding the central cylinder. The last one is associated with a blood stream in the central vessel’s lumen ($\Omega_{BF}$). The next external covering pipe corresponds to ESG ($\Omega_{ESG}$). Then, the sequence of covers is as follows: the endothelium ($\Omega_{EN}$), the basal lamina ($\Omega_{BL}$), the rest space in the tunica intima ($\Omega_{rsTI}$), IEL ($\Omega_{IEL}$), the tunica media ($\Omega_{TM}$), and the tunica adventitia ($\Omega_{TA}$). They are created by placing the appropriate cylinders with the same central axis orientation and initial coordinates. These basic geometric elements make up the assortment of inserted forms that are ultimately utilized to establish the modelling areas (Figure 2).

All domains of the 3D digital phantom were created using the Geometry Module of COMSOL Multiphysics ver. 5.5. To obtain an appropriate domain, a Boolean difference operation was applied to the covering cylinders and the inserted cylinders, consequently

$$\Omega_{domain}=\Omega_{cylinder}^{\mathrm{cov}ering}\backslash \Omega_{cylinder}^{inserted}$$

Each final domain is a pipe that maintains a fixed thickness ($h_{index}$, see Table 1), corresponding to the difference between the radii of the cylinders.

**Figure 3:**
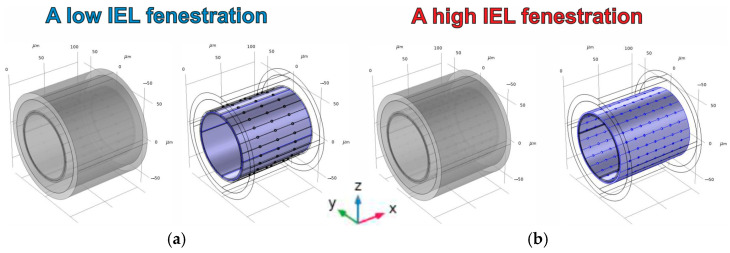
Different cases of IEL fenestration. In the 3D digital phantom of an arteria, two variants of IEL permeability are supposed to be considered. They are formed by the number of penetrating pores in IEL. (**a**) The panel illustrates the case of a low number of pores in IEL. The left part implies all structures of the phantom, and the right part shows a highlighted IEL domain with an explicit indication of pores. (**b**) The panel represents the same phantom with a high number of pores. All indications and properties are the same as in Figure 3a.

The pores in the IEL domain were created as a Boolean difference between $\Omega_{IEL}^{Initial}$ and $\Omega_{pores}$:

$$\Omega_{IEL}=\Omega_{IEL}^{Initial}\backslash \Omega_{pores};\;\Omega_{pores}={\left\{\Omega_{pore}^{i}\right\}}_{i=1}^{N}$$

where $\Omega_{pore}^{i}$ is the cylinder with the diameter $d_{pore}$ and the axis orthogonal to the central axis of $\Omega_{IEL}^{Initial}$. To deal with the difference in IEL fenestration, two cases are considered in the phantom design. The first one is associated with a low IEL fenestration, accomplished as three symmetric rings of pores penetrating IEL (Figure 3a). The direction of penetration is orthogonal to the central axis of the IEL pipe’s domain. The case of high fenestration is related to seven symmetric rings of pores (Figure 3b).

The diffusion properties of the pores are not the same as those of the main IEL body. It is supposed that the permeability of IEL is very low, and on the contrary the diffusion ion the pores is characterized by the same diffusion coefficient as in the other parts of the vessel’s wall.

All longitudinal boundaries of the pipes ($\partial \Omega_{BF}^{L},\partial \Omega_{ESG/EN/BL/rsTI/IEL/TM}^{L_{in/out}},\partial \Omega_{TA}^{L_{in}}$) form the internal surfaces in the phantom. In the case of convectional reaction–diffusion, there are no boundary conditions on them. The exception is the external boundary of the tunica adventitia ($\partial \Omega_{TA}^{L_{out}}$) because this surface is a part of the external boundary of the phantom (∂Ω). However, the conditions are set up in the case of convection because there is a separate area for the blood flow and interstitial fluid. The side boundaries ($\partial \Omega_{BF/ESG/EN/BL/rsTI/IEL/TM/TA}^{S_{in/out}}$) have defined conditions for both diffusion and convection.

The geometrical parameters used in the creation of the phantom are represented in Table 1. It is remarkable that the tunica intima consists of several anatomical structures, which are considered separately. For example, the endothelium, the basal lamina, and IEL form separate domains. At the same time, the size of the tunica intima is larger than the sum of their thicknesses. The difference is considered as a rest part of the tunica intima ($\Omega_{rsTI}$).

A small artery, characterized by an 80 μm diameter, exemplifies the 3D digital phantom that has been created in the present study. The geometrical parameters of the final domains have been verified with a precision level of 0.04%. The reference values of the volumes and the surface areas used for further modelling are represented in Table 2. They can be used for recalibrating the fluxes and adjusting the model to accommodate different geometric dimensions of the phantom.

### 2.2. Calculations of the Convectional Field in the Phantom

Having designed the 3D digital phantom of the arteria as described above, one is able to evaluate the field of convection. This problem is shared in two parts. Initially, a distribution of velocities has to be obtained for a blood flow in a lumen domain ($\Omega_{BF}\cup \Omega_{ESG}$). Then, the convection will be estimated in the blood vessel wall.

To consider the velocity field in $\Omega_{BF}\cup \Omega_{ESG}$, a boundary problem for the Navier–Stokes equation for non-Newtonian liquid should be numerically solved [27].

(1) $$\begin{array}{l} \rho \frac{\partial \vec{u}}{\partial t}+\rho \left(\vec{u}\cdot \nabla \right)\vec{u}=-\nabla p+\nabla \left(\mu \left(\left|\dot{\gamma }\right|\right)\left(\nabla \vec{u}+{\left(\nabla \vec{u}\right)}^{T}\right)\right);\\ \dot{\gamma }=2\varepsilon =\nabla \vec{u}+{\left(\nabla \vec{u}\right)}^{T},\;\left|\dot{\gamma }\right|=\sqrt{2\left(\varepsilon :\varepsilon \right)};\\ \nabla \vec{u}=0; \end{array}$$

where $\vec{u}$ is the velocity vector along the coordinate system; ε denotes strain-rate tensor; p, t and ρ are pressure, time, and the fluid density, respectively. There is no slip on the boundary of the arteria lumen. In the present study, the Carreau model has been used for description of the non-Newtonian fluid flow dynamics viscosity μ on shear rate $\left|\dot{\gamma }\right|$ [28]. According to the Carreau model, the dynamics viscosity is described by the following expression:

(2) μγ˙=μ∞+μ0−μ∞1+λγ˙2n−12

where $\mu_{\infty }$ connotes a constant limit value when blood is treated as a Newtonian fluid, $\mu_{0}$ is the blood viscosity at a zero shear rate, λ is the time constant associated with the viscosity that changes with the shear rate, and n is an index parameter [29]. The parameters’ values used for the modelling and the method of Equations (1) and (2) solving is described in detail in previous studies [27,28].

**Figure 4:**
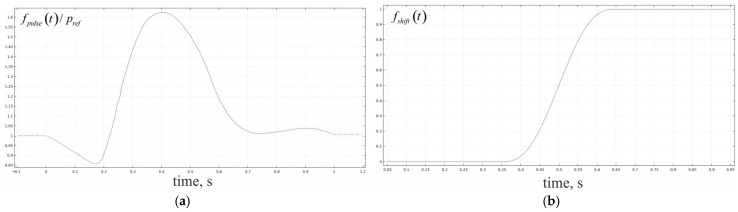
The dimensionless functions, which are used for the governing of the blood pressure time dependence. The considered cases correspond to a pulse wave (**a**) and a shift in pressure (**b**).

The boundary conditions are applied to the side boundaries of the lumen domain ($\Omega_{BF}\cup \Omega_{ESG}$). They can form different types of pressure dependence on time. The first variant corresponds to a pulse wave of pressure.

(3) $$\begin{array}{l} p_{in}=p_{0}+\frac{\Delta p_{80\mu m}^{\min}\cdot f_{pulse}\left(t\right)}{2p_{ref}},\;\;\vec{r}\in \left(\partial \Omega_{BF}^{S_{in}}\cup \partial \Omega_{ESG}^{S_{in}}\right)\\ p_{out}=p_{0}-\frac{\Delta p_{80\mu m}^{\min}\cdot f_{pulse}\left(t\right)}{2p_{ref}},\;\;\vec{r}\in \left(\partial \Omega_{BF}^{S_{out}}\cup \partial \Omega_{ESG}^{S_{out}}\right) \end{array}$$

where $\Delta p_{80\mu m}^{\min}$, $p_{0}$ and $p_{ref}$ are the parameters determined according to experimental data. The function $f_{pulse}\left(t\right)$ is the time dependence which is shown in Figure 4a. It is supposed that the changes in the inside vessel blood pressure will appear as pulse waves, and the main characteristics are repeatable in time. This is why only one period can be considered for the analysis.

The other variant describes a shift in pressure from the low to the high level in the blood vessel:

(4) $$\begin{array}{l} p_{in}=p_{0}\left(1+\left(\alpha_{80\mu m}-1\right)\cdot f_{shift}\left(t\right)\right)+g\left(t\right),\;\;\vec{r}\in \left(\partial \Omega_{BF}^{S_{in}}\cup \partial \Omega_{ESG}^{S_{in}}\right)\\ p_{out}=p_{0}\left(1+\left(\alpha_{80\mu m}-1\right)\cdot f_{shift}\left(t\right)\right)-g\left(t\right),\;\;\vec{r}\in \left(\partial \Omega_{BF}^{S_{out}}\cup \partial \Omega_{ESG}^{S_{out}}\right)\\ g\left(t\right)=\left(\frac{\Delta p_{80\mu m}^{\min}}{\beta_{80\mu m}^{\prime }}+\left(\frac{\Delta p_{80\mu m}^{\max}}{\beta_{80\mu m}^{\prime \prime }}-\frac{\Delta p_{80\mu m}^{\min}}{\beta_{80\mu m}^{\prime }}\right)\cdot f_{shift}\left(t\right)\right); \end{array}$$

The function $f_{shift}\left(t\right)$ is the time dependence for such kind changes, and it is shown in Figure 4b. Several parameters are the same ones as in Equation (3); however, the additional values are used to adjust the pressure to the experimental data. The set of parameters validating conditions in Equations (3) and (4) is represented in Table 3.

Having solved the boundary problem for Equations (1) and (2), including the condition in Equation (3) or (4), one should obtain the function of the wall pressure and the shear stress. These functions are necessary to evaluate the convectional velocity field in the arterial wall and to create the dependence of eNOS activity, respectively.

The convectional velocity field in the blood vessel wall is evaluated according to Darcy’s law with the following boundary conditions:

(5) $$\begin{array}{l} \vec{u}=-\frac{\kappa_{medium}^{EN/TI/IEL/TM/TA}}{\mu_{medium}}\nabla p_{medium},\;\nabla \vec{u}=0,\;\\ \vec{r}\in \Omega_{EN}\cup \left(\Omega_{TI}\backslash \Omega_{IEL}\right)\cup \Omega_{IEL}\cup \Omega_{TM}\cup \Omega_{TA};\\ p_{medium}=f_{pulse/shift}^{wall}\left(t\right),\;\vec{r}\in \partial \Omega_{EN}^{L_{in}};\\ p_{medium}=p_{out},\;\vec{r}\in \partial \Omega_{TA}^{L_{out}}\cup \partial \Omega_{TA}^{S_{in}}\cup \partial \Omega_{TA}^{S_{out}}; \end{array}$$

**Figure 5:**
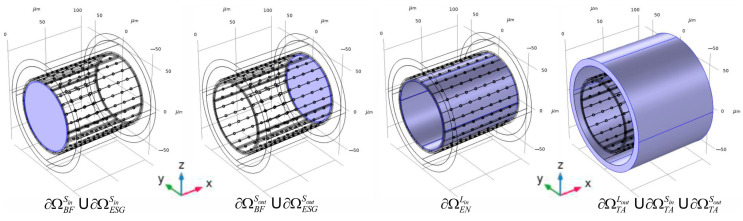
Indication of the boundaries used for the boundary conditions in Equations (3) and (4). The represented case corresponds to a high fenestration of IEL.

The values of permeability and the dynamic viscosity of the medium are represented in Table 3. The time dependence of pressure ($f_{pulse/shift}^{wall}\left(t\right)$) is obtained after solving the boundary problem for the Navier–Stokes equation. The curve shape is identical to that depicted in Figure 4 for the cases of pulse and shift. The boundaries used in Equations (3)–(5) are shown in Figure 5.

Having obtained the convectional velocity field, one is able to consider the gradients of NO in a blood vessel wall. A special feature of the problem is that the production of NO appears in a narrow domain ($\Omega_{EN}$), with further distribution into the inner domain ($\Omega_{BF}\cup \Omega_{ESG}$) characterized by a high velocity rate of convection and the direct forming of a concentration gradient in the external domains with a low convectional velocity.

### 2.3. Evaluation of the NO Gradients Using a Boundary Problem for the Convectional Reaction–Diffusion Equation

The concentration of NO is spatially distributed around the endothelium in a blood vessel wall and inside a vessel lumen. Generally, one can assume that the diffusion of NO occurs symmetrically and isotopically. It means that the values of diagonal elements of the diffusion tensor will be the same inside any considered domain. However, they can be different depending on the type of domain. For example, the changes in the structure and viscosity of ESG cause alteration in the diffusion coefficient, i.e., the amplitude value of diagonal diffusion tensor elements. The previously obtained convectional velocity fields must also be considered. Finally, the consumption and production rates in the domain are the essential processes forming the spatial–temporal gradients of NO in the phantom. Contemplating the claims mentioned above, the convectional reaction–diffusion equation can be represented in the following form:

(6) $$\begin{array}{l} \frac{\partial c_{NO}\left(\vec{r},t\right)}{\partial t}=\nabla \cdot \left(D\cdot \nabla c_{NO}\left(\vec{r},t\right)\right)-\vec{u}\left(\vec{r},t\right)\cdot \nabla c_{NO}\left(\vec{r},t\right)+f_{con}\left(c_{NO}\left(\vec{r},t\right),\vec{r}\right),\;\vec{r}\in \Omega ;\\ \nabla ={\vec{e}}_{x}\frac{\partial }{\partial x}+{\vec{e}}_{y}\frac{\partial }{\partial y}+{\vec{e}}_{z}\frac{\partial }{\partial z} \end{array}$$

where D, $\vec{u}$ and $f_{con}\left(c_{NO}\left(\vec{r},t\right),\vec{r}\right)$ are the diffusion tensor, the convection velocity and the reaction rate of NO production/consumption, respectively. The elements of diffusion tensors can be represented as the production of the mean diffusion coefficient ($D_{NO}^{/ESG_{i}/IEL}$) and isotropy matrix (n = 3) [27]. The last summand on the right side of Equation (6) is essentially varied among the domains of the phantom. The main difference is observed between $\Omega_{BF}$, $\Omega_{EN}$, and the rest part of Ω. In the central part of the lumen blood vessel domain ($\Omega_{BF}$), NO is scavenged by hemoglobin contained in red blood cells [33]. This process is considered without the possible non-enzymatic NO production released by these cells. In the endothelium occur both the production of NO provided by eNOS activity and its consumption mediated by NO binding to sGC [34]. The rate of eNOS is shear stress-dependent, and after the blood flow changes, it can vary in time [35]. In the abluminal region, NO is assumed to be consumed by a second-order reaction characterized by an overall reaction rate constant that accounts for the contribution of various NO decomposition mechanisms and reactions with other species in the smooth muscle tissue [36]. Taken together, the final form of the third term of the right part of Equation (6) can be represented in the following expression:

(7) $$f_{con}\left(c_{NO}\left(\vec{r},t\right),\vec{r}\right)=\left\{\begin{array}{l} -\left(k_{tissue}\cdot c_{NO}\left(\vec{r},t\right)+k_{ablum}\cdot c_{NO}{\left(\vec{r},t\right)}^{2}\right),\;\vec{r}\in \Omega \backslash \left(\Omega_{BF}\cup \Omega_{EN}\right);\\ -\left(k_{HB}\cdot c_{NO}\left(\vec{r},t\right)+k_{ablum}\cdot c_{NO}{\left(\vec{r},t\right)}^{2}\right),\;\vec{r}\in \Omega_{BF};\\ \alpha_{eNOS}\cdot J_{ref}^{NO}\cdot \frac{f_{shear\;stress}\left(t\right)}{\tau_{ref}}-k_{tissue}\cdot c_{NO}\left(\vec{r},t\right),\;\vec{r}\in \Omega_{EN}; \end{array}\right.$$

Equation (6) with expression (7) should be accompanied by the initial and the boundary conditions, forming a boundary problem. At the initial moment of time, the concentration of NO is equal to the base level in all domains.

(8) $$c_{NO}\left(\vec{r},t\right){|}_{t=0}=c_{NO}^{base},\;\vec{r}\in \Omega ;$$

For the boundaries, there several types of used conditions. For the lumen domain and ESG ($\Omega_{BF}$, $\Omega_{ESG}$), the conditions are orientated according to the convection blood flow. For the inflow side boundary, the condition is the Danckwerts flux, and for the outflow side boundary, it is assumed that the main part of NO is removed by the blood stream. The open boundary conditions are applied to the external border surfaces of the tunica adventitia. For all other cases, the Dirichlet boundary condition is used. Taking into account the assumption made above, one can write the following expression:

(9) $$\begin{array}{l} \vec{n}\cdot \left(-D\cdot \nabla c_{NO}\left(\vec{r},t\right)+\vec{u}\cdot c_{NO}\left(\vec{r},t\right)\right)=\vec{n}\cdot \left(\vec{u}\cdot c_{NO}^{base}\right);\;\vec{r}\in \partial \Omega_{BF}^{S_{in}}\\ \vec{n}\cdot \left(D\cdot \nabla c_{NO}\left(\vec{r},t\right)\right)=0;\;\vec{r}\in \partial \Omega_{BF}^{S_{out}}\\ if\;\vec{n}\cdot \vec{u}\geq 0\Rightarrow \vec{n}\cdot \left(D\cdot \nabla c_{NO}\left(\vec{r},t\right)\right)=0;\;\vec{r}\in \partial \Omega_{TA}^{L_{out}}\cup \partial \Omega_{TA}^{S_{in}}\cup \partial \Omega_{TA}^{S_{out}}\\ if\;\vec{n}\cdot \vec{u}<0\Rightarrow c_{NO}\left(\vec{r},t\right)=c_{NO}^{base};\;\vec{r}\in \partial \Omega_{TA}^{L_{out}}\cup \partial \Omega_{TA}^{S_{in}}\cup \partial \Omega_{TA}^{S_{out}}\\ c_{NO}\left(\vec{r},t\right)=c_{NO}^{base};\;\vec{r}\in \partial \Omega \backslash \left(\partial \Omega_{BF}^{S_{in}}\cup \partial \Omega_{BF}^{S_{out}}\cup \partial \Omega_{TA}^{L_{out}}\cup \partial \Omega_{TA}^{S_{in}}\cup \partial \Omega_{TA}^{S_{out}}\right) \end{array}$$

$\vec{n}$ is a unit normal vector corresponding to an appropriate surface. The parameters used in Equations (6)–(9) are represented in Table 4. The variation in the diffusion coefficients in the different domains reflects the properties of the blood vessel wall and ESG.

The evaluation of the boundary problem for Equations (6) and (7) with the initial conditions (8) and the boundary conditions (9) make it possible to obtain the spatial–temporal distribution of NO concentration in all domains of the considered phantom.

### 2.4. The Numerical Method of the Boundary Problem Evaluation Used in the Present Study

The considered boundary problems for both convectional flows and reaction–diffusion were numerically evaluated using the finite element method (FEM) in COMSOL Multiphysics software version 5.5. The phantoms were created for the cases of both low and high IEL fenestrations. The finalized geometry for the low fenestration of IEL has 81 domains, 826 boundaries, 1824 edges, and 1016 vertices. The finalized geometry for the high IEL fenestration includes 177 domains, 1802 boundaries, 3952 edges, and 2168 vertices. The evaluation of the convectional velocity field and NO concentration gradients was obtained in each domain of the phantom using the discretization of the geometry into small units of simple shapes, referred to as mesh elements. There several meshes with different characteristics were built for this study. The description of the meshes is represented in Table 5. The boundary problems for the Navier–Stokes equation (Equations (1)–(3)/(4)) and the convectional reaction–diffusion (Equations (6)–(9)) in the phantoms were numerically evaluated using Meshes #1 and #3, which correspond to a low IEL and a high IEL fenestration, respectively. For the numerical solution of the boundary problem according to Darcy’s law (Equarion (5)), Meshes #2 and #4 were used for to a low IEL and a high IEL fenestration, respectively.

The calculations were fulfilled using the Physical Modules of COMSOL Multiphysics 5.5, including Laminar Flow (spf), Darcy’s Law (dl), and the Transport of Diluted Species in Porus Media (tds). The study was structured in three sequential phases, employing a time interval from 0 to 1 s with increments of 0.01 s.

## 3. Results

The obtained results of the present study consist of two parts. In the beginning, it is necessary to evaluate the convection velocity field in every domain of the phantom. Furthermore, it is necessary to estimate the shear stress and wall pressure in $\Omega_{BF}$ and $\partial \Omega_{EN}^{L_{in}}$. These values will be the functions of time. Then, non-steady state gradients of NO can be appraised in the blood flow and in the blood vessel wall. Notably, the variations in diffusion coefficients within ESG and the degree of IEL fenestration are crucial in dictating how these structures facilitate the distribution of NO in the vessel wall, provided that the time dynamic of eNOS activity is fixed. In the next sections, the numeric evaluation of the convectional velocity field and the spatial–temporal NO concentration distribution will be sequentially considered.

### 3.1. Evaluation of the Convectional Velocity Field in the Phantom

**Figure 6:**
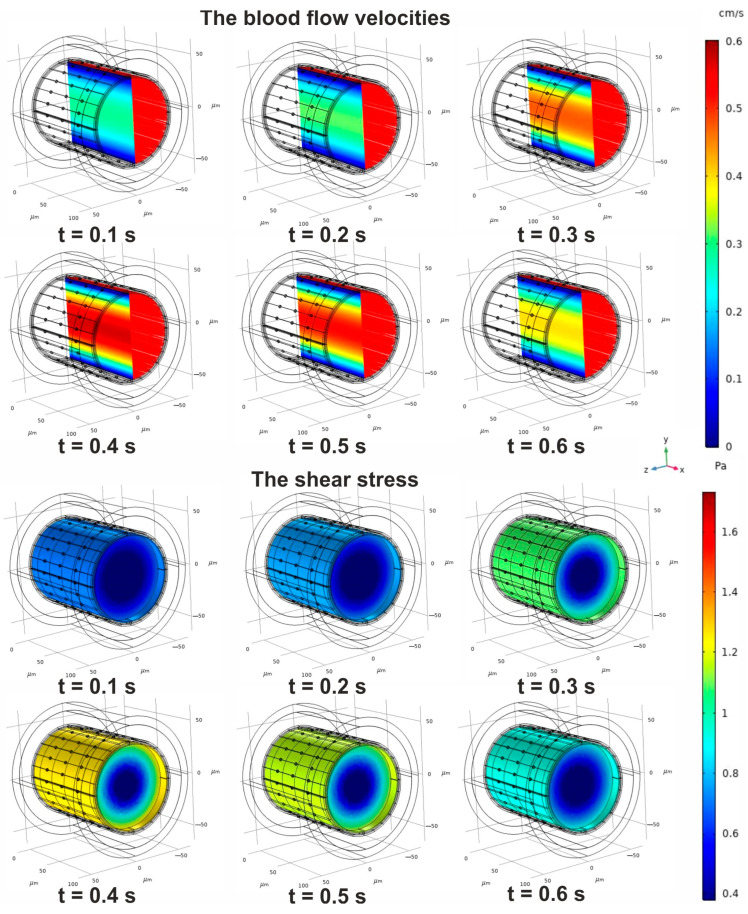
The convectional variables for $\Omega_{BF}$ and $\Omega_{ESG}$ domains in the case of a pulse wave of pressure. The upper panel represents the distribution of the blood flow velocities in the central cut plane of the domains. The red colour behind the plane indicates the streamlines of velocities in the domains. The low panel represents the shear stress in the domains and $\partial \Omega_{EN}^{L_{in}}$. The represented geometry corresponds to a low level of IEL fenestration.

The convectional velocity field is obtained in all domains of the phantom. Nevertheless, the first result should be the solution of the Navier–Stokes equation with the boundary conditions because to solve the problem for Equation (5), one needs to obtain a numeric estimation of the wall pressure on $\partial \Omega_{EN}^{L_{in}}$. The time-dependent images for the velocities and the shear stress in the blood stream ($\Omega_{BF}$) and ESG ($\Omega_{ESG}$) are represented in Figure 6. Both variables are governed by the function $f_{pulse}\left(t\right)$, with an increasing/decreasing amplitude in time. One can observe a distribution of velocity rates in the blood circulation and ESG, characterized by a typical increase in amplitude near the central axis. Indeed, the time-dependent behaviour of the shear stress aligns with the governing pressure function, and the same results can be gained in the case of $f_{shift}\left(t\right)$.

**Figure 7:**
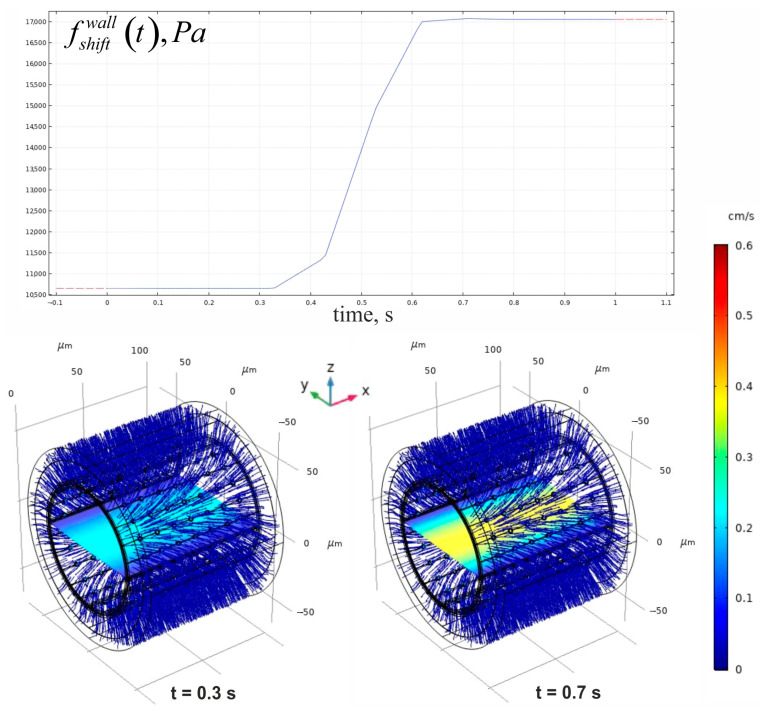
The convectional variables for $\Omega_{BF}$ and $\Omega_{ESG}$ domains in the case of a shift in pressure. In the upper panel, $f_{shift}^{wall}\left(t\right)$ is shown. The bottom segment of the figure exposes two sets of velocity distributions associated with both low and high pressures in the vessel. The distributions are illustrated by the pattern of the central plane in $\Omega_{BF}$ and $\Omega_{ESG}$ domains. The blue arrows that outline the regions depict the streamlines of convective velocities based on Darcy’s law in the blood vessel wall. The represented geometry corresponds to a high level of IEL fenestration.

Having used the solution of the boundary problem based on Equations (1) and (2), the explicit forms for both $f_{shear\;stress}\left(t\right)$ and $f_{pulse/shift}^{wall}\left(t\right)$ can be retrieved more easily. The numerical results of the wall pressure over time are leveraged to make further evaluations of the convection velocity field according to Darcy’s law in the blood vessel wall. Since the pressure demonstrates a smooth temporal dependence on both blood flow and wall convection, it is feasible to consider only two states that align with low and high pressures, respectively. According to this assumption, the time points of 0.3 s and 0.7 s have been identified as significant. The examples of such patterns are represented in Figure 7.

As noted earlier, the dependence of wall pressure on time is determined by the pressure function applied in the boundary conditions of the Navier–Stokes equation problem. The amplitude of blood flow velocity varies by more than 60% in the examined cases. The evaluated parameters of the conventional velocity field allow for an assessment of how blood circulation affects the development of a NO gradient.

### 3.2. Evaluation of a Spatial-Temporal Distribution of NO Concentrations Considering the Effects of Blood Flow Velocities, ESG Permeability and IEL Fenestration

This section focuses on the quantitative assessment of the boundary problem formulated to the conventional reaction–diffusion equation and how the characteristics of the phantom medium influence it. The amplitude of the NO concentration gradient is established through multiple conditions that shape its characteristics. They are the activity of eNOS, the barriers for NO diffusion and a convectional flow in the blood stream. Although the synthesis of NO typically draws the most interest, other factors mentioned above significantly contribute as well. The physical and chemical properties of the environment, in conjunction with the geometric design of the domains, can either hinder or facilitate the spread of NO. The primary aspect to investigate in terms of its effect is the convection velocity field.

#### 3.2.1. Forming a Gradient of NO Concentrations Under Different Blood Flow Rates

**Figure 8:**
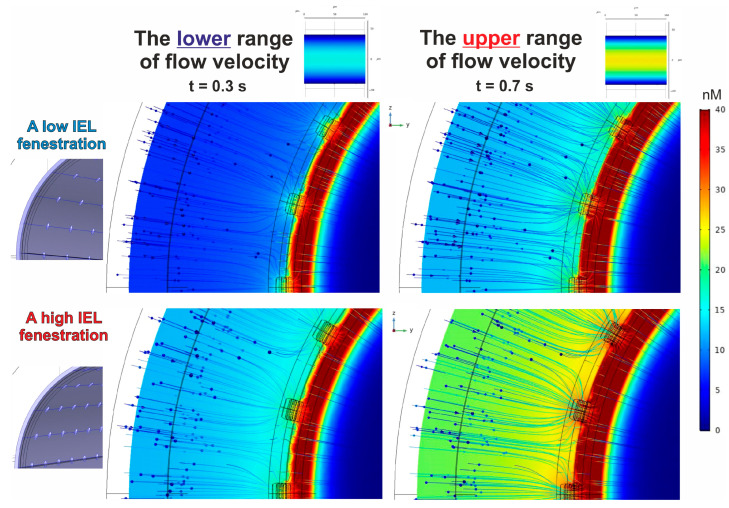
The patterns of NO concentration gradient in the blood flow and the blood vessel wall. Blue arrows indicate the total NO concentration flux. The parameters of eNOS activity and the scale amplitude coefficient of the diffusion tensor in ESG are $\alpha_{eNOS}=1$ and $D_{NO}^{ESG_{0.5}}$, respectively. A lower blood flow velocity condition shows a maximum concentration of 46 nM, whereas a gradient under the high blood flow rate has a maximum of 68 nM. The thresholds are identical for both varieties of IEL fenestration.

As highlighted earlier, the analysis focuses solely on two types of conventional velocity fields. One value represents the minimum average velocity of blood flow, while the other indicates the maximum flow rate for the specified vessel diameter. The patterns of NO concentration gradients are represented in Figure 8.

It is clear that a rise in blood flow velocity leads to a greater amplitude of the NO concentration gradient when the barrier conditions remain constant. Remarkably, this phenomenon is maintained across different forms of IEL fenestration.

#### 3.2.2. The Effect of IEL Pore Density and Diffusion Permeability of ESG on the NO Concentration Gradients

**Figure 9:**
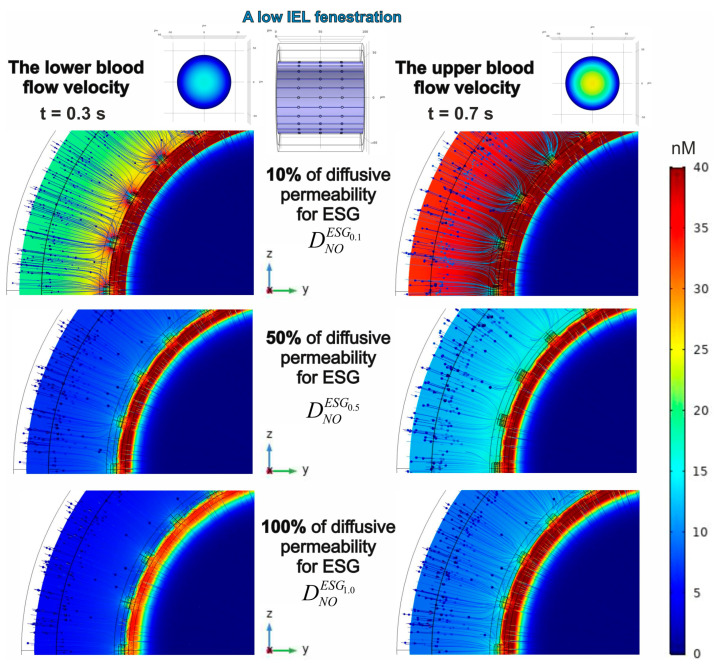
The case of low IEL fenestration and the influence of the diffusive permeability of ESG on the formation of the NO concentration gradient in the blood flow and the blood vessel wall. Blue arrows indicate the total NO concentration flux. The left set of patterns corresponds to a low blood flow velocity, and the right one shows the gradients for a high blood flow velocity. The parameters of eNOS activity are $\alpha_{eNOS}=1$.

**Figure 10:**
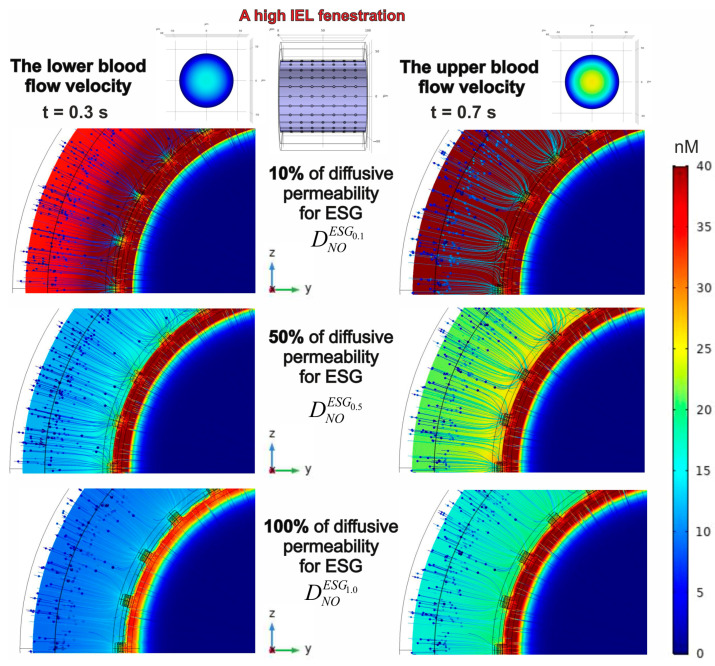
The case of high IEL fenestration and the influence of the diffusive permeability of ESG on the formation of the NO concentration gradient in the blood flow and the blood vessel wall. The parameters and indicators remain identical to those presented in Figure 9.

The results presented above reveal the NO gradient acquired with the fixed parameters of ESG. An analysis of two variations in IEL porosity showed that an increase in pore quantity results in a stronger NO gradient. This result suggests that a reduction in barrier function affects the gradient that develops behind the barrier. In analyzing this concept, several variants of ESG diffusive permeability were considered. It is shown that an increase in the diffusion coefficient of the glycocalyx leads to a notable decrease in the amplitude of the NO concentration gradient, assuming the amplitude of eNOS activity remains unchanged. The effect occurs when there is a presence of both low (Figure 9) and high (Figure 10) fenestration in the IEL.

In general, one can see that, in most instances, the endothelium ($\Omega_{EN}$) contains the greatest concentration of NO exceeding 40 nM, although there is one notable exception. If the diffusive permeability of ESG aligns with that of other domains in the phantom (except for $\Omega_{IEL}$), the amplitude of NO spatial concentration distribution decreases to 35 nM under a low blood flow (Figure 9 and Figure 10; bottom/left). Although the distribution patterns of NO concentrations are wholly shaped by the fenestration characteristics of the IEL, the enhanced blood flow velocity rather than the increased quantity of pores in the IEL restores the NO levels in the endothelium (Figure 9 and Figure 10; bottom/right).

One needs to notice that the diffusion barriers have different effects on NO concentration gradients. While the permeability of ESG affects both the strength of the gradient and its peak intensity, the fenestration of IEL primarily influences the distribution of values represented by colours, without impacting the highest values. An illustration of this is that when the ESG diffusion coefficient rises from 10% to 50% of its typical levels, the peak concentration of NO drops from 191 nM to 68.2 nM under conditions of a high blood flow (t = 0.7 s). However, the alterations in IEL pore structure result in an insignificant variation between these measurements, with 191 nM (68.2 nM) corresponding to a lower pore count in IEL and 189 nM (68.4 nM) reflecting a higher pore count. The heightened rate of eNOS, resulting from high pressure and a high blood flow velocity, reveals an equivalent effect.

A reduction in the diffusive permeability of ESG significantly enhances the NO concentration gradient’s amplitude, regardless of the blood flow velocities and the number of pores in the IEL. It is also remarkable that under any circumstances, the central lumen consistently shows low concentrations of NO.

#### 3.2.3. The Relationship Between eNOS Activity Levels and the Concentration Distribution of NO

**Figure 11:**
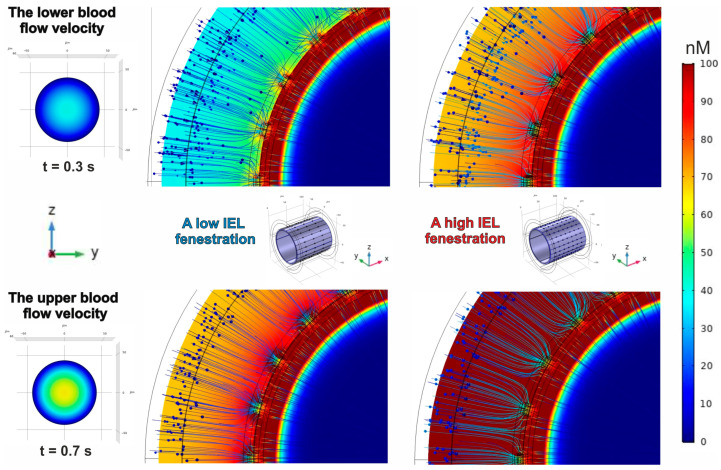
The patterns of the NO concentration gradient in the blood flow and the blood vessel wall. Blue arrows indicate the total NO concentration flux. The parameters of eNOS activity and the scale amplitude coefficient of the diffusion tensor in ESG are $\alpha_{eNOS}=5.46$ and $D_{NO}^{ESG_{0.5}}$, respectively. A lower blood flow velocity condition shows a maximum concentration of 250 nM, whereas a gradient under the high blood flow rate has a maximum of 373 nM. The thresholds are identical for both varieties of IEL fenestration.

An increase in the coefficient $\alpha_{eNOS}$ will result in heightened NO concentrations throughout every domain of the phantom. This parameter demonstrates the degree of expression of the eNOS gene, along with other mechanisms that regulate its activation. The results of the evaluation of NO concentration gradients under enhanced $\alpha_{eNOS}$ are represented in Figure 11.

Undoubtedly, the impact of reducing diffusive barriers on the gradients of NO concentration remains consistent with what was demonstrated above. One should notice that the fluctuations in blood flow rates lead to a 49% enhancement of the NO gradient, while an increase in IEL fenestration shows a 79% rise in the amplitude of the concentration spatial distribution.

Thus, the findings from the modelling distinctly illustrate how conventional blood flow, the level of IEL fenestration, and the diffusive permeability of ESG contribute to the establishment of a gradient in NO concentration.

## 4. Discussion

In the context of the formation of NO concentration gradients, it is important to highlight several fundamental points. They are (i) a relation between blood flow dynamics and NO concentration gradients; (ii) the mechanisms of IEL and ESG effects, and (iii) the forming of NO concentration amplitude.

The primary aspect is the role of blood flow in the initiation and evolution of the aforementioned spatial–temporal distribution of NO. The used approach makes it possible to consider both the pulse and shift dynamics of pressure changes in the arteria. Certainly, it is logical to acknowledge the explicit nature of pulse dynamics in relation to major arteries, including the aortas. With the decline in pulse pressure associated with smaller arterial diameters [42], it is reasonable to infer that the actual pressure in the considered geometry will remain stable, while it can be modified from low to high values through the shifting process. Attention should be directed towards the idea that a pulse alteration can generally be modelled as a two-step shift from a lower pressure state to a higher one and back again. The outcome of the present study represents velocity distribution, wall pressure, and shear stress at each point of a blood vessel lumen and its surface. Since the pressure demonstrates a smooth temporal dependence on both blood flow and wall convection, it is adequate to investigate the NO gradients for only two variations of blood circulation and blood vessel wall convection. The low velocity and the high velocity were chosen to form the convectional velocity fields in this study. One should notice that the highest and lowest velocities derived from the model align well with the results obtained from experiments for arteries with such diameters [30]. In particular, the average value of a low velocity in $\Omega_{BF}\cup \Omega_{ESG}$ is as high as 0.124 cm/s, and the average value of a high velocity in the same domains is equal to 0.200 cm/s. The blood flow velocities observed in blood vessels with an 80 μm diameter are 0.129 cm/s for the lower threshold and 0.200 cm/s for the upper threshold [30].

Thus, several molecular physical mechanisms play a role in how variations in the velocity of blood flow produce non-homogeneous gradients of NO concentrations. First, the process of eliminating produced nitric oxide is achieved through a direct convection movement. Second, it is essential that the process of convection works alongside a significant level of NO consumption, facilitated by its binding to hemoglobin. Third, elevated blood pressure leads to an increase in shear stress levels, subsequently stimulating the activity of eNOS. Among the processes described above, the primary impact of changes in blood flow velocity on the NO concentration gradient is driven by the activation of eNOS, which is stimulated by shear stress increase. This phenomenon is most evident in the blood flow scenarios being analyzed.

Another aspect affecting the concentration of NO in the system is the barriers created by IEL and ESG. The architecture of blood vessels is notably perceived as a factor in the actions of NO, with ESG being highlighted as a potential player in the role of this mediator within the vascular system [43]. Notably, the integrity of the ESG structure is consistently viewed as a crucial element in the development of nitric oxide concentration gradients. Glycocalyx degradation ablated the shear stress-stimulated NO production [44], and the characteristics of the inner surface of a blood vessel can be altered in relation to shear stress via a mechanism that depends on ESG viscosity [45]. Moreover, the endothelium can be protected against the lipopolysaccharide-induced increase in cell barrier permeability and NO production by preserving the integrity of glycocalyx [46]. Experiments carried out in living rats indicate that the glycocalyx is essential for controlling the changes in hemodynamics within their arteries [47]. In the context of ischemia and reperfusion, which usually cause endothelium–glycocalyx disruption [48], NO contributes to heart protection by alleviating the no-reflow phenomenon in coronary circulation and preventing leakage and edema in coronary vessels, thanks to the preservation of the endothelial glycocalyx [49].

The chemical constituents of ESG play a vital role in the production of NO through the action of eNOS [50,51]. Using a fluorescence microscopy approach, it was shown that the glycocalyx senses and transduces vertical mechanical stretch to produce NO depending on the presence of both heparan sulphate (HS) and hyaluronic acid (HA) in ESG, as their removal leads to a significant decrease in NO production [52]. It is remarkable that low shear stress can trigger the activation of the enzyme responsible for HA degradation, which results in a reduction in NO production levels [53]. Furthermore, experiments conducted at varying blood flow rates indicate that the degradation of ESG, due to the elimination of HS, leads to a decrease in NO production [15]. However, evidence from experiments indicates that although HS and HA play a clear role, they are not directly involved in the production of NO triggered by shear stress [54].

The experimental observations considered above affirm that the state and arrangement of ESG’s influence the production of NO by the endothelium. However, the main attention is directed towards how changes caused by mechanical forces affect eNOS activity and contribute to the formation of shear stress phenomena. Nevertheless, the results of the present study reveal that the gradient of NO can be altered by changes in the diffusive permeability of ESG, which arise due to viscosity fluctuations. The decline in NO concentration at both low and high shear stress conditions can be attributed to its ability to surpass the diffusion barrier beneath the endothelium in a vascular lumen. Furthermore, NO diffusing particles reach the area where there is a strong convection flow and extreme consumption. This observation provides an alternative viewpoint on the experimental findings, focusing on the physical characteristics of the vessel structural layer. A decline in the distribution of the NO concentration resulting from damaged ESG, coupled with facilitated diffusion, can occur alongside the variations in shear stress effects on eNOS.

The function of IEL in the establishment of a NO concentration gradient is identical to that of the glycocalyx [55]. It forms the external border for the tunica intima, providing the metabolites diffusion through the network of pores. The fenestration of IEL plays a crucial role in establishing concentration gradients for both large and small molecules. For example, the ATP concentration distribution depends strongly on the IEL pore structure because ATP fluid-phase transport is dominated by diffusion emanating from the fenestral pores [14]. The quantity of pores in the IEL fluctuates over time and it is never assumed to be stable [56]. Moreover, some authors have demonstrated in animals that the number of fenestrae of IEL increases during normal postnatal development, and that they are larger when the blood flow is artificially augmented in systemic arteries [26]. A chronic increase in arterial blood flow leads to gaps in the IEL widening as intercommunicating circumferential and longitudinal luminal depressions, occupying more than 60% of the lumen surface [57]. Based on the analysis presented in this research, one could assert that the greater density of pores within the IEL boosts the range of spatial and temporal variations in NO levels. This effect arises from a heightened permeability of NO to the environment, occurring at a consistent (rather than elevated) consumption rate.

Thus, both ESG and IEL act as impediments to the spread of NO generated in the middle layer of the tunica intima. This case reveals the competition between two diffusive barriers in the development of a gradient for NO concentration. Under circumstances of diminished IEL fenestration and $D_{NO}^{ESG_{1.0}}$, NO is preferably circulated throughout a domain characterized by the intense convective flow and high levels of consumption [58]. This results in the depletion of the endothelial domain. The growing quantity of pores in the IEL will help reestablish equilibrium and redirect the diffusion towards the blood vessel wall, leading to the saturation of the endothelium. A higher ESG viscosity, which means lower diffusive permeability, creates a significant barrier for nitric oxide to access the lumen region and subsequently vanish.

Another noteworthy consideration is the diversity of NO concentration present in the walls of blood vessels and in different types of tissues. In reality, it is not standardized and can shift considerably depending on functional aspects and internal influences. The highest concentration of NO is ultimately influenced by the rate of eNOS, which is regulated by shear stress and the expression of genes. The represented study incorporates both factors into the time variability of eNOS, which is not a fixed value. Earlier research demonstrated that the amplitude of the gradient in NO concentration is directly linked to the metabolic flux in a linear manner [59,60]. Certainly, in the present study, the level is explicitly governed by $\alpha_{eNOS}\cdot J_{ref}^{NO}$. This parameter can be easily adapted to fine-tune the strength of the NO gradient for various scenarios. Due to a straightforward form of reliance, all previously mentioned effects will remain intact, with only the concentration level being altered. There are several studies indicating that the physiological signalling concentrations of NO are between 100 pM and 5 nM, and this is 100- to 1000-fold higher than the dissociation constant for NO bound to sGC [61]. In different areas of the brain, the recorded evoked NO signals in the cerebellum yielded values of between 2 nM and 2 μM, whereas values of 0.12 μM to 2 μM were reported in the hippocampus after maximal NMDA stimulation [62]. Indeed, in this type of tissue, nNOS can be considered as an additional source of NO [63]. However, one also observes a comparable diversity in the measured levels of NO within both vascular and perivascular regions. The reported values range from 4 nM [35] and about 200 to 1000 nM under control conditions [64]. The methodology employed in this research allows for the assessment of NO concentration variations across various amplitudes, spanning a diverse spectrum of values. Besides a basic enhancement in eNOS rate, the inherent physical properties of IEL and ESG induce significant changes in the spatial distribution of NO concentration. It is tempting to suppose that alterations in the permeability characteristics of blood vessel components often coincide with the modulation of eNOS, and they are crucial elements of the physiological system that regulates NO levels.

The essential characteristic of the introduced method is its ability to be implemented on a network of arterial trees. Furthermore, the approach to designing 3D phantoms allows for the direct assessment of pathological states by analyzing blood flow interruptions. It may be achievable through the addition of further domains into $\Omega_{BF}\cup \Omega_{ESG}$ that act like a clot. The assessment of injury to a blood vessel’s wall can also be evaluated by examining the geometric alterations within the relevant areas. The characteristics of this model will serve as a useful tool for both theoretical and experimental research in combinatory studies.

## 5. Conclusions

In the present study, a mathematical model of convectional reaction–diffusion designed in 3D digital phantom for a small arteria is applied to the evaluation of non-steady-state NO concentration gradients. The role of two diffusive barriers surrounding the endothelium producing NO was theoretically proven. At any given rate of eNOS, an increase in the fenestration of IEL and a decrease in the diffusive permeability of ESG lead to a notable rise in NO concentration in the vascular wall. The theoretical modelling allows us to understand that changes in ESG content can trigger not only the modification of eNOS activity through shear stress transduction but also that the viscosity of ESG can directly facilitate the formation of a NO gradient. The alterations in pore count in IEL and the viscosity of ESG are considered to be involved in the physiological and pathological regulation of NO concentrations.

## Figures and Tables

**Table 1:** The set of geometrical parameters used for 3D digital phantom creation.

Geometrical Parameter	Value (μm)	Literature and Comments
$L_{vessel}$	100	[20] ^1^
$R_{0}$	40	[20] ^2^
$h_{wall}$	51.3	[21] ^3^
$h_{ESG}$	1	[13,22]
$h_{EN}$	1	[23]
$h_{TI}$	10.39	[24,25] ^4^
$h_{IEL}$	1.38	[26]
$h_{TM}$	40.78	[24,25]
$h_{TA}$	24.79	[24,25]
$d_{pore}$	2.8	[14]
$L_{p-p}$	5.6	[14]

^1^ The length was chosen to avoid shaping features of a blood vessel. ^2^ The radius corresponds to a small arteria. ^3^ The thickness of the blood vessel wall was calculated according to the following relation [21]: $h_{wall}/R_{0}=a\cdot \exp\left(b\cdot R_{0}\right)+c\cdot \exp\left(d\cdot R_{0}\right)$ where *a* = 0.2802, *b* = −5.053 1/cm, *c* = 0.1324, *d* = −0.1114 1/cm. ^4^ Thickness values for the various tunica layers are derived from known experimental data.

**Table 2:** The size of the domains included in the 3D digital phantom. The values were evaluated using the Geometry Module of COMSOL Multiphysics ver. 5.5.

Structure	Value	Domains and Surfaces
A blood stream		
Volume	477,630.0 μm^3^	$\Omega_{BF}$
Surface area	34,054.0 μm^2^	$\partial \Omega_{BF}^{L}\cup \partial \Omega_{BF}^{S_{in}}\cup \partial \Omega_{BF}^{S_{out}}$
ESG		
Volume	24,821.0 μm^3^	$\Omega_{ESG}$
Surface area	50,128.0 μm^2^	$\partial \Omega_{ESG}^{L_{in}}\cup \partial \Omega_{ESG}^{L_{out}}\cup \partial \Omega_{ESG}^{S_{in}}\cup \partial \Omega_{ESG}^{S_{out}}$
The endothelium		
Volume	25,437.0 μm^3^	$\Omega_{EN}$
Surface area	51,397.0 μm^2^	$\partial \Omega_{EN}^{L_{in}}\cup \partial \Omega_{EN}^{L_{out}}\cup \partial \Omega_{EN}^{S_{in}}\cup \partial \Omega_{EN}^{S_{out}}$
IEL		
Volume	36,920.0 μm^3^	$\Omega_{IEL}$
Surface area	52,717.0 μm^2^	$\partial \Omega_{IEL}^{L_{in}}\cup \partial \Omega_{IEL}^{L_{out}}\cup \partial \Omega_{IEL}^{S_{in}}\cup \partial \Omega_{IEL}^{S_{out}}$
Pores (a low IEL fenestration)		
Volume	557.19 μm^3^	$\Omega_{pores}$ ^1^
Surface area	1539 μm^2^	$\partial \Omega_{pores}^{L}\cup \partial \Omega_{pores}^{S_{in}}\cup \partial \Omega_{pores}^{S_{out}}$
Pores (a high IEL fenestration)		
Volume	1300.1 μm^3^	The designations are the same as for a low IEL fenestration
Surface area	3591.1 μm^2^	-
The tunica media		
Volume	405,440.0 μm^3^	$\Omega_{TM}$
Surface area	70,644.0 μm^2^	$\partial \Omega_{TM}^{L_{in}}\cup \partial \Omega_{TM}^{L_{out}}\cup \partial \Omega_{TM}^{S_{in}}\cup \partial \Omega_{TM}^{S_{out}}$
The tunica adventitia		
Volume	296,200.0 μm^3^	$\Omega_{TA}$
Surface area	81,648.0 μm^2^	$\partial \Omega_{TA}^{L_{in}}\cup \partial \Omega_{TA}^{L_{out}}\cup \partial \Omega_{TA}^{S_{in}}\cup \partial \Omega_{TA}^{S_{out}}$

^1^ The designation indicates the united set of domains, which includes the number or pores penetrating IEL. It is possible to obtain the parameters for each pore separately, but it has not been performed because of the symmetric properties of the model.

**Table 3:** The parameters used for forming the boundary condition of the Navier–Stokes equation and evaluation of the convection velocity field according to Darcy’s law.

Parameter	Value	Literature and Comments
$p_{ref}$	10,666 Pa	[30]
$p_{0}$	10,661 Pa	$\mathrm{The}\;\mathrm{calculated}\;\mathrm{on}\;\mathrm{the}\;\mathrm{base}\;\mathrm{of}\;p_{ref}$ minus shift in pressure according to Poiseuille’s law
$\Delta p_{80\mu m}^{\min}$	3.875 Pa	This value is calculated according to Poiseuille’s law for the minimal blood flow velocity
$\Delta p_{80\mu m}^{\max}$	5.99 Pa	The same calculation as in a previous row, but it has been performed for the maximal blood flow velocity
$\alpha_{80\mu m}$	1.6	An empiric coefficient
$\beta_{80\mu m}^{\prime }$	2.6	An empiric coefficient
$\beta_{80\mu m}^{\prime \prime }$	2.7	An empiric coefficient
$p_{out}$	3999.7 Pa	[31]
$\mu_{medium}$	0.72 × 10^−3^ g/(mm∙s)	[32]
$\kappa_{medium}^{EN}$	4.32 × 10^−15^ mm^2^	[32]
$\kappa_{medium}^{TI}$	2.00 × 10^−10^ mm^2^	[32]
$\kappa_{medium}^{IEL}$	4.392 × 10^−13^ mm^2^	[32]
$\kappa_{medium}^{TM}$	2.00 × 10^−12^ mm^2^	[32]
$\kappa_{medium}^{TA}$	5.061 × 10^−11^ mm^2^	The average value

**Table 4:** The parameters of diffusion, production, and consumption of NO in the considered part of a blood flow and a blood vessel wall. The diffusion coefficients correspond to the scale amplitude coefficient of the diffusion tensor (see text).

Parameter	Value	Literature and Comments
$D_{NO}$	3.3 × 10^−5^ cm^2^/s	[37,38]
$D_{NO}^{ESG_{1.0}}$	3.3 × 10^−5^ cm^2^/s	The assumed value ^1^
$D_{NO}^{ESG_{0.5}}$	1.65 × 10^−5^ cm^2^/s	[39]
$D_{NO}^{ESG_{0.1}}$	3.3 × 10^−6^ cm^2^/s	[40]
$D_{NO}^{IEL}$	3.18 × 10^−11^ cm^2^/s	[31]
$k_{tissue}$	0.01 1/s	[34,41]
$k_{ablum}$	0.05 1/(μM∙s)	[36,41]
$k_{HB}$	1230 1/s	[33]
$J_{ref}^{NO}$	150 μM/s	[35]
$\tau_{ref}$	2.4 Pa	[35]
$\alpha_{eNOS}$	1 or 5.46	An empiric coefficient ^2^

^1^ One alternative could lead to the considered diffusion coefficient being as high as it is for the rest of the medium. Then, $D_{NO}^{ESG_{1.0}}=D_{NO}$. ^2^ It is assumed that the activity of eNOS can be varied by gene expression. To taking this into account, one includes an empiric coefficient into Equation (7) for the NO production flux.

**Table 5:** The description of mesh characteristics used in the model. The represented values are the number of appropriate elements.

Meshes	Domain Elements	Boundary Elements	Edge Elements
Mesh #1 *	9,948,439	1,361,050	19,861
Mesh #2	602,764	130,592	8118
Mesh #3	10,361,539	1,407,458	26,664
Mesh #4	743,741	161,988	12,210

* The Mesh #1 and Mesh #3 are specified in the Mesh Module of COMSOL Multiphysics 5.5 as “Finer” types. Whereas Mesh#2 and Mesh #4 related to “Normal” types in that classification.

## Data Availability

The original contributions presented in this study are included in the article. Further inquiries can be directed to the corresponding author(s).

## Abbreviations

The following abbreviations are used in this manuscript:

NO	Nitric oxide
NOS	Nitric oxide synthase
nNOS	The neuronal isoenzyme of nitric oxide synthase
iNOS	The inducible isoenzyme of nitric oxide synathase
eNOS	The endothelial isoenzyme of nitric oxide synthase
ROS	Reactive oxygen species
sGC	Soluble guanylyl cyclase
ONOO–	Peroxynitrite
IEL	The internal elastic lamina
ESG	A thin layer of endothelial surface glycocalyx
GACs	Glycosaminoglycans
FEM	The finite element method
HS	Heparan sulphate
HA	Hyaluronic acid (hyaluronan)
ATP	Adenosine triphosphate
